# Unit resection of buccal squamous cell carcinoma: Description of a new surgical technique

**DOI:** 10.18632/oncotarget.14191

**Published:** 2016-12-25

**Authors:** Zhen-Hu Ren, Zhao-Jian Gong, Han-Jiang Wu

**Affiliations:** ^1^ Department of Oral and Maxillofacial surgery, Second Xiangya hospital of Central South University, Changsha, Hunan, 410011, China; ^2^ Department of Oral Maxillofacial-Head and Neck Oncology, Ninth People's Hospital, Shanghai Jiao Tong University School of Medicine, Shanghai 200011, China

**Keywords:** buccal squamous cell carcinoma, muscle anatomy, surgical treatment, unit resection, survival

## Abstract

This study characterized the infiltration of primary tumors along the muscles, fascia and spaces of the maxillofacial region in buccal squamous cell carcinoma (BSCC) and suggested a new surgical strategy that is suitable for most stages. Based on the anatomic characteristics and infiltration of the primary tumor a new surgical approach - unit resection buccal surgery (URBS) - was developed. We evaluated this new surgical strategy, across a cohort of 127 BSCCs: 60 cases treated with URBS and 67 cases treated with conventional surgery. Notably there was no statistical difference in the clinicopathological variables between the two groups. After initial treatment with curative intent, the patients were regularly followed-up with clinical examination and imaging. URBS proved suitable for almost all stages of BSCC, and was particularly advantageous for advanced stages of BSCC. At 2 years post-treatment, the rates of overall survival were 83.3% in the URBS group and 60.1% in the conventional surgery group, respectively (hazard ratio 0.38; 95% CI 0.20 to 0.75; P=0.005). Similarly, the rates of disease-free survival were 76.6% and 51.9% in the URBS group and the conventional surgery group, respectively (hazard ratio 0.42; 95% CI 0.23 to 0.75; P=0.003). The principles of URBS are suitable for almost all stages of BSCC, especially advanced stages. URBS may improve the prognosis of BSCC patients.

## INTRODUCTION

Oral squamous cell carcinoma (OSCC) is the sixth most common cancer in the world and accounts for nearly 3% of all cancers [1–5]. Because of the high prevalence of betel quid chewing, the incidence of buccal squamous cell carcinoma (BSCC), together with other cancers of the oral cavity, have been increasing in China and other regions and countries of Asia. OSCC remains one of the major malignancies in these areas [6] and surgery is still the most important modality of treatment [7–10]. Although significant advances have been made in the prevention, diagnosis, and therapy, in the past 20 years only modest progress has been made in improving the survival rates in patients with progressive or metastatic disease. Even among early stage OSCCs with adequate resection margins, the recurrence rate ranges from 33.3% to 57.7% [11]. However, studies exploring surgical techniques for BSCC are very limited, especially regarding removal of the primary tumor.

Removing the primary tumor with a wide margin of normal tissue is the most important principle of surgical oncology [3]. However, what constitutes a sufficiently appropriate margin, particularly in BSCC, is fundamentally unclear. While the generally accepted standard is to remove a circumferential margin of 1.5 to 2 cm [12] around the primary tumor, a significant number of patients with negative margins develop recurrence at the primary site.

In an effort to improve survival and present recurrence in patients with BSCC, we developed a new surgical strategy that is suitable for BSCC patients and performed a preliminary evaluation of this surgical approach, named unit (subunit) resection buccal surgery (URBS). This research may be helpful in the management of BSCC patients undergoing radical surgery.

## RESULTS

**Figure 1:**
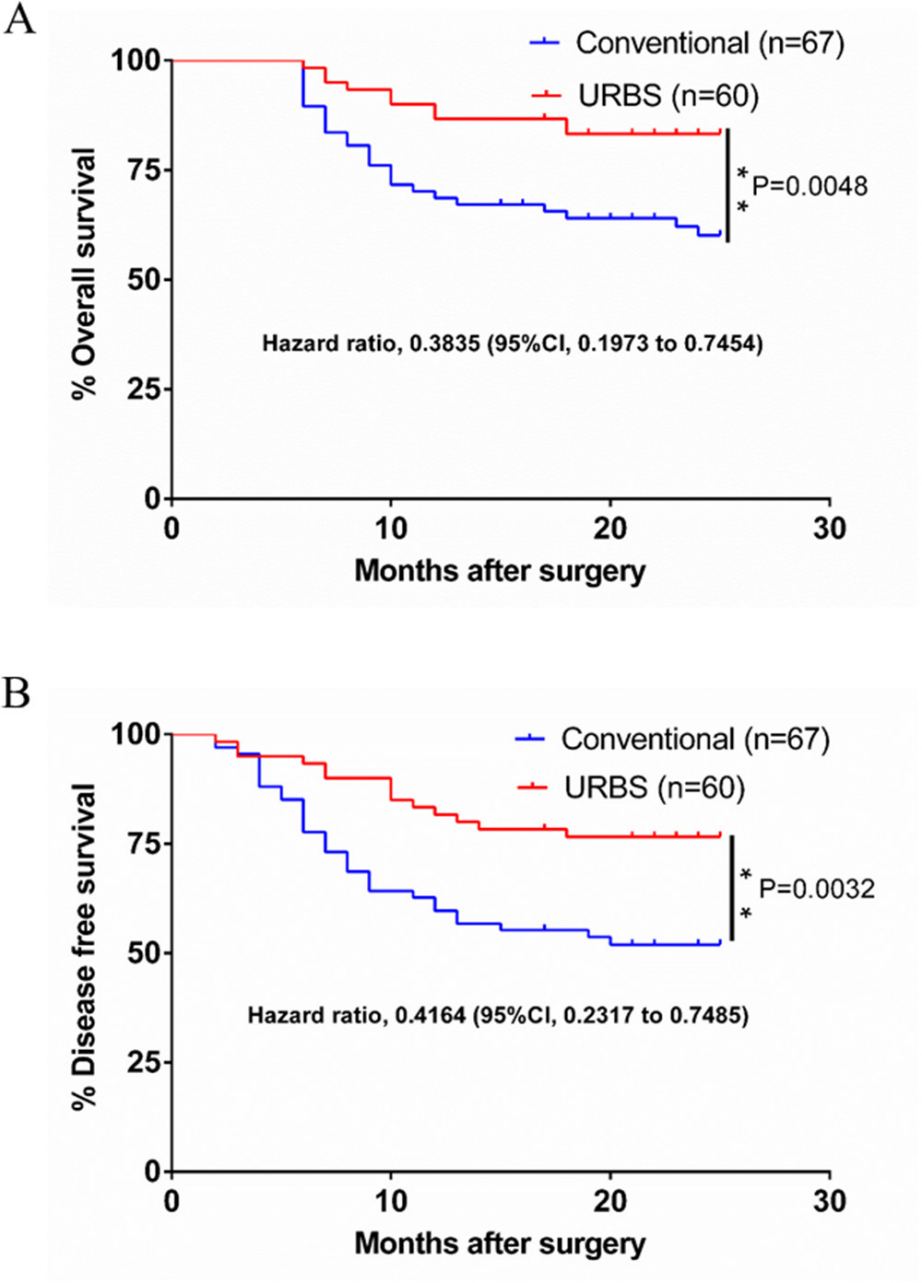
The survival of the URBS group and the conventional surgery group **A**. Overall survival at 2 years after operation was 83.3% and 60.1% in the URBS group and the conventional surgery group, respectively (hazard ratio 0.38; 95% CI 0.20 to 0.75; P=0.005). **B**. Disease-free survival was 76.6% and 51.9% in the URBS group and the conventional surgery group, respectively (hazard ratio 0.42; 95% CI 0.23 to 0.75; P=0.003).

The patient cohort in our study included 127 patients: 96 male and 31 female. The age range was 25-76 (median age 54.5). The cohort was divided into two groups: a URBS group composed of 60 cases, and a conventional group composed of 67 cases. The median follow-up was 20.9 months (range 2–26). At 2 years post resection, the rates of overall survival were 83.3% and 60.1% in the URBS group and conventional surgery group, respectively (hazard ratio 0.38; 95% CI 0.20 to 0.75; P=0.005). The rates of disease-free survival were 76.6% and 51.9% in the URBS group and the conventional surgery group, respectively (hazard ratio 0.42; 95% CI 0.23 to 0.75; P=0.003) (Figure 1). Objective functional evaluation suggested all patients could at least eat soft diet and had acceptable communication skills. The quality of life results demonstrated that chewing, swallowing, and speech recovered perfectly in 80% of these patients in both groups. Mouth opening was more than 3.5 cm in greater than 90% of the patients in both groups (Table 1).

**Table 1:** Functional results after URBS or conventional surgery for patients with BSCC

Groups	Number of cases	Chew function, n (%)	Swallowing function, n(%)	Voice, n(%)	Appearance, n(%)	Mouth opening, n(%)
URBS	60	Normal: 51 (85.0%) Only soft food: 8 (13.3%) Unable to chew: 1 (1.7%)	Normal: 58 (96.7%) Liquids: 2 (3.3%) Solids: 0 (0%)	Normal: 53 (88.3%) Barely able to communicate: 7 (11.7%) Unable to communicate:0 (0%)	Good: 6 (10.0%) Acceptable: 42 (70.0%) Unacceptable:12 (20.0%)	≥3.5 cm: 55 (91.7%) 1.5–3.5 cm: 3 (5.0%) <1.5 cm: 2 (3.3%)
Conventional surgery	67	Normal: 56 (83.6%) Only soft food: 10 (14.9%) Unable to chew: 1 (1.5%)	Normal: 64 (95.5%) Liquids: 2 (3.0%) Solids: 1 (1.5%)	Normal: 59 (88.1%) Barely able to communicate: 8(11.9%) Unable to communicate:0 (0%)	Good: 12(17.9%) Acceptable: 47 (70.1%) Unacceptable:8 (11.9%)	≥3.5 cm: 56 (83.6%) 1.5–3.5 cm: 8 (11.9%) <1.5 cm: 3 (4.5%)

Characteristics of enrolled patients are shown in Table 2. The relationship between different groups and clinicopathologic variables are shown in Table 3.

**Table 2:** Characteristics of the patients enrolled in this study (n=127)

Characteristics	Value
Age	54.5(25-76)
Sex	
Male	96 (75.6)
Female	31(24.4)
T stage	
T1	47(37.0)
T2	48(37.8)
T3	19(15.0)
T4	13(10.2)
Differentiation	
Well differentiated	84(66.1)
Moderate differentiated	33(26.0)
Poorly differentiated	10(7.9)
Metastasis lymph nodes	
Positive	41(32.3)
Negative	86(67.7)
Groups	
URBS	60(31.9)
Conventional surgery	67(68.1)

**Table 3:** Association between the patient's clinicopathologic characteristics and groups in 127 BSCC patients

Clinicopathologic features	No.	Groups		P
		URBS	NC	
Gender				0.496
Male	96	47	49	
Female	31	13	18	
Age, years				0.459
<60	70	31	39	
≥60	57	29	28	
Smoking				0.417
Yes	47	20	27	
No	80	40	40	
Drinking				0.362
Yes	40	16	24	
No	87	44	43	
Tumor stage				0.136
1-2	95	41	54	
3-4	32	19	13	
Lymph node metastasis				0.843
+	41	20	21	
-	86	40	46	
Histological type				0.516
Poor	10	3	6	
Well- Moderate	117	57	61	
Postoperative radiotherapy				0.684
+	12	5	7	
-	115	55	60	
Postoperative chemotherapy				0.861
+	9	4	5	
-	118	56	62	

## DISCUSSION

BSCC is a cancer associated with a low rate of contralateral neck recurrence, high rate of locoregional recurrence, and poor overall survival [6, 7]. Wide resection of the tumor with ipsilateral neck dissection is the primary modality of treatment [13, 14]. While surgical resection and treatment of BSCC has improved, prognosis remains poor for patients, in part due to the recurrence rate [15]. Buccal carcinoma has a higher recurrence rate and a lower 5-year survival than other oral cancers [6, 16]. Studies have suggest that the absecne of relatively fixed anatomic barriers that do not have enough limiting effect on primary tumor growth and spread account for the recurrence rate [17]. As such, the clinical classification of buccal carcinoma lesions, the in-depth study of its local and adjacent anatomical regions, and discussion regarding modifications to the methodology by which buccal carcinoma is resected is critically important. The main focus of this study is to propose compartmental resection and the ability to achieve pathologically negative margins.

Importantly, the reasons for the high locoregional failure need to be further explored. Possible explanations include inadequate treatment and intrinsic tendency to an aggressive nature, however, the lack of an anatomic barrier in the cheek has also been previously cited [18]. Proposed and applied compartment surgery improves the local control rate of buccal carcinoma. While we believe that the interpretation which Trivedi et al [1] made regarding compartment surgery is incomplete, the concept of compartment surgery cannot explain buccal carcinoma surgical characteristics accurately. We believe that unit resection reveals the extent of tumor infiltration more accurately, and thus permits a more thorough removal of tumor cells to achieve the goal of radical treatment.

The feasibility and safety of the procedure is demonstrated by the results in this series. While some scholars may question whether such a large unit resection may damage local function of patients, we believe even after conventional surgery, the residual portion of these structures do not maintain any function, as all their attachments to the mandible are sectioned, and the residual portions are involved by a massive fibrosis. In addition, through interview and functional evaluation of patients we found no more dysfunction in patients who underwent unit resection as compared with patients that underwent conventional surgery.

For greater tissue defects of primary sites due to radical resection of buccal carcinoma, we should choose flaps with large supplies of tissue [19], such as the latissimus dorsi flap [20], or anterolateral thigh muscle flap. Anterolateral thigh muscle flap (ALT) has certain advantages in defect reconstruction for its rich tissue mass and plasticity [21]. For example, ALT may be prepared according to the flap needs of different thickness in different regions to a single island or multi-island flap with a single pedicle, and may also carry the muscles, fascia, fat flap, etc [21, 22]. 1. The non-through defect: routinely prepare a single lobe flap to repair intra-oral defects. For a case of zygomatic expand resection on postgena, de-epithelialized flaps and muscle flaps can be used to fill the dead space in inferior zygomatic region. 2. Through-and-through defect: prepared a double-island free flap with single pedicle, those two islands are used to repair intra-oral or extra-oral defects an each island has its own independent skin perforator blood supply. Vermilion elastic flap can be used to repair defects of the lip due to radical resection of pregena carcinoma. Folded single island flap repair should be avoided in through-and-through defect [21]. If a double-island flap with a single pedicle can not be prepared due to vascular causes, a folded portion of single lobe should be placed on postgena to make better shape of the pregena and mouth corner.

In conclusion, surgical resection of BSCC represents a challenging clinical problem due to inadequate and inaccurate preoperative assessment of disease spread and difficult surgery. Unit resection to remove all the possible infiltration paths of cancer cells is feasible and improves deep soft tissue margin control without adding functional morbidity. Application of the principles of URBS to the treatment of BSCC proved suitable for almost all stages of the disease, especially the advanced stage of BSCC.

## MATERIALS AND METHODS

### Inclusion and exclusion criteria

We designed and implemented a clinical observational study and a retrospective cohort analysis to achieve the objectives of this study. This study was approval by the Institutional Review Board (IRB) of the Second Xiangya Hospital at Central South University, China. From March 2011 to January 2014, 127 patients with BSCC were treated in the Department of Oral and Maxillofacial Surgery of the Second Xiangya Hospital. The inclusion criteria included: 1) patients had to have been diagnosed of tongue squamous cell carcinoma by pathological examination; and 2) patients had not received surgical treatment, radiotherapy, chemotherapy, or other treatment prior to surgery. Patients were excluded from this study if they had distant metastases. A standard pre-operative assessment was performed after patients were admitted to hospital, including laboratory examination, chest X-ray, head and neck computed tomography (CT), head and neck magnetic resonance imaging (MRI), and PET-CT (if necessary). Head and neck CT or MRI was used for the preliminary evaluation of pattern of spread and size of BSCC primary tumor and cervical lymph nodes.

### Surgical technique

Upon completion of surgical resection, guided by the preoperative imaging data, we carefully dissected the tumor along multiple axes. We measured the size of the tumor and the tumor thickness and determined the infiltrate law of buccal carcinoma along the muscles, fascia, and spaces of maxillofacial region. We summarized the infiltrate law of buccal carcinoma along the muscles, fascia and spaces of maxillofacial region in BSCC, and created a new surgical strategy.

**Figure 2:**
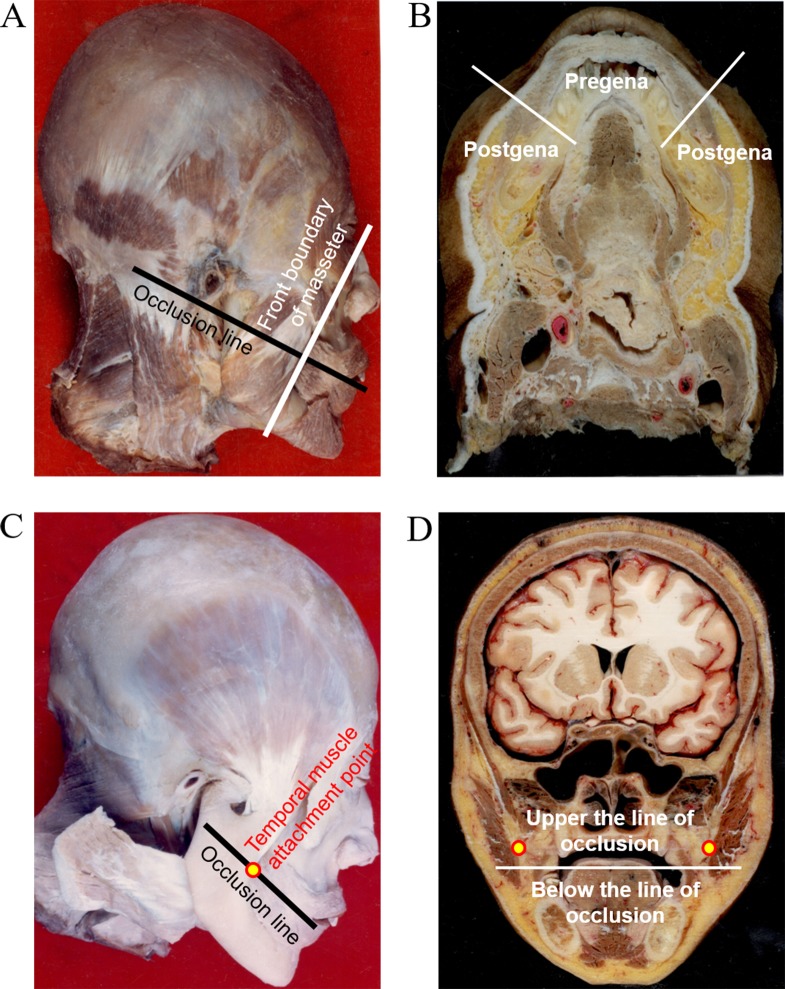
The buccal was divided into 4 parts **A**. We divided the cheek into four parts using the anterior border of masseter and the line of occlusion as the boundary. **B**. Layers of tissue from buccal mucosa to skin are mucosa, submucosa and loose connective tissue, mimetic muscles and skin, respectively in Pregena, and the buccal mucosa, submucosal connective tissue, anterior border of mandibular ramus, masseter muscle, parotid gland and skin, respectively in Postgena. **C**. Temporal muscle attachment points are in the line of occlusion. **D**. The position relationship between temporal muscle and the line of occlusion.

According to the specific anatomical characteristics of each region, we divided the cheek into four parts (Figure 2A): 1. Pregena: anatomically, the pregena is bounded posteriorly by the anterior border of masseter, anteriorly by the upperlip and underlip of the medial mucosa. Layers of tissue from the buccal mucosa to the skin are mucosa, submucosa, and loose connective tissue, mimetic muscles and skin, respectively (Figure 2B). Tumors can invade the thin buccinator muscle and spread to the skin direction more easily. 2. Postgena; the postgena is bounded anteriorly by the anterior border of the masseter, and posteriorly by the pterygomandibular fold. From the inside out in this area are the buccal mucosa, submucosal connective tissue, anterior border of mandibular ramus, masseter muscle, parotid gland, and skin (Figure 2B). The region has sufficient thickness to get enough perimeter when removing the tumor. But this area has a close relationship to the medial pterygoid, lower parts of the anterior temporal muscle and buccopharyngeal fascia, which facilitates tumor spread. 3. The buccal mucosa can be divided into upper and lower regions by the line of occlusion. (Figure 2C). Structures such as the medial pterygoid, buccal fat pad, maxillary tuberosity and fibers of temporal muscle that have close relationship with postgena, also enhance tumor spread along those structures or spaces (Figure 2D). 4. The buccal mucosa below the line of occlusion can also be divided into pregena and postgena by the anterior border of the masseter. Anatomical characteristics were the same as pregena and postgena that were mentioned above in the first point and the second point (Figure 2C and 2D). However, this area has a close relationship to the mandible but not a particularly close relationship with the temporalis and the medial pterygoid. In this regard, tumor cannot spread to the inferior zygomatic region easily.

**Figure 3:**
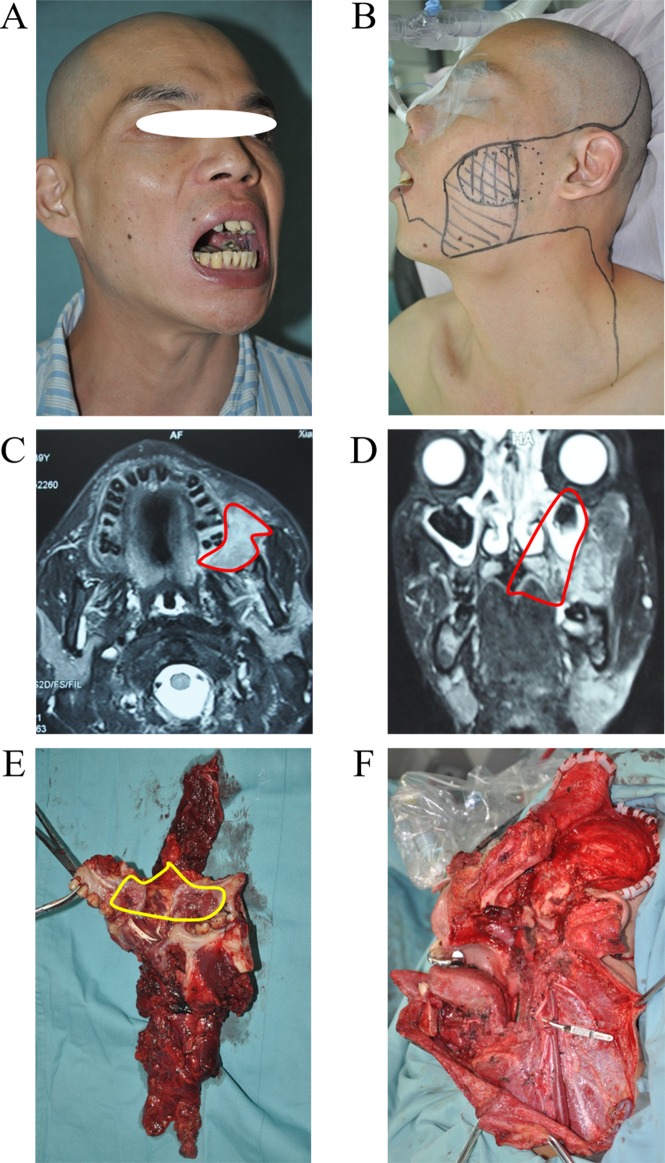
Cancer extends along the direction of the masticatory muscle fibers This cases had URBS. From the samples, we can clearly see that cancer cells are infiltrating along the direction of the muscle fibers of temporalis and medial pterygoid.

**Figure 4:**
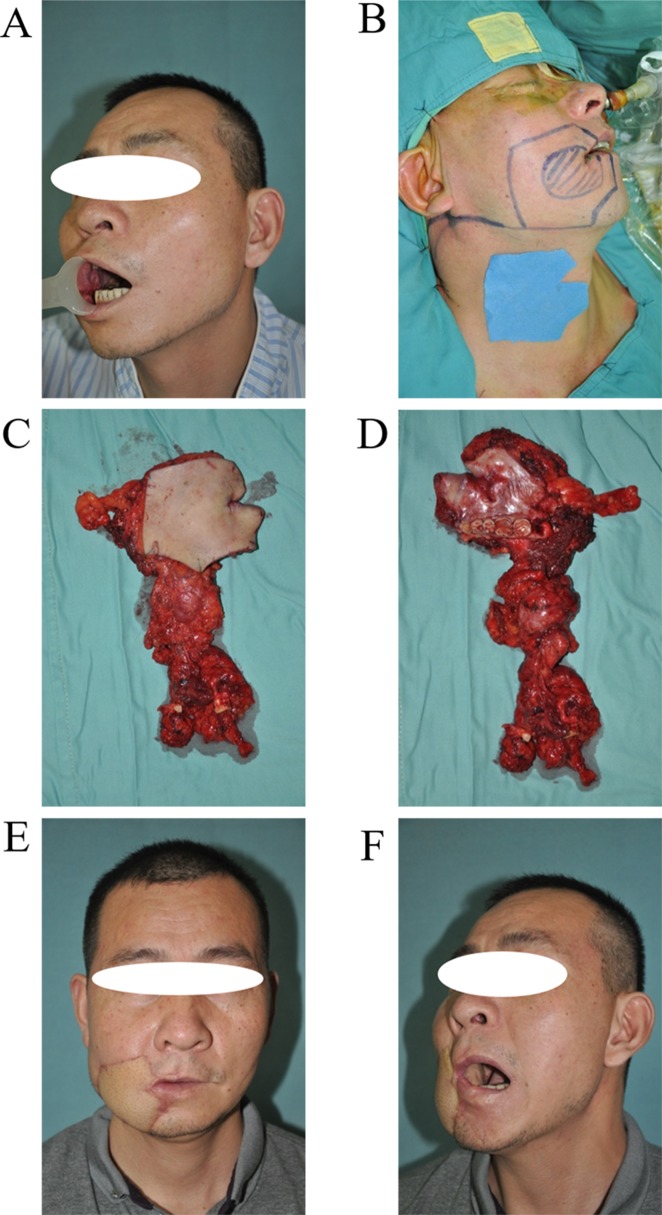
URBS of cancer located in the pregena **A**. Cancer located in the pregena, tumor invasion to the lips. **B**. Tumor incision design and the template of free flap for reconstruction. **C**. The primary tumor and neck dissection tissue (from skin). **D**. The primary tumor and neck dissection tissue (from mucosa). **E**. The photo of a patient 1 year postoperatively. **F**. The photo of the patient with mouth open (mouth opening is 3 cm).

**Figure 5:**
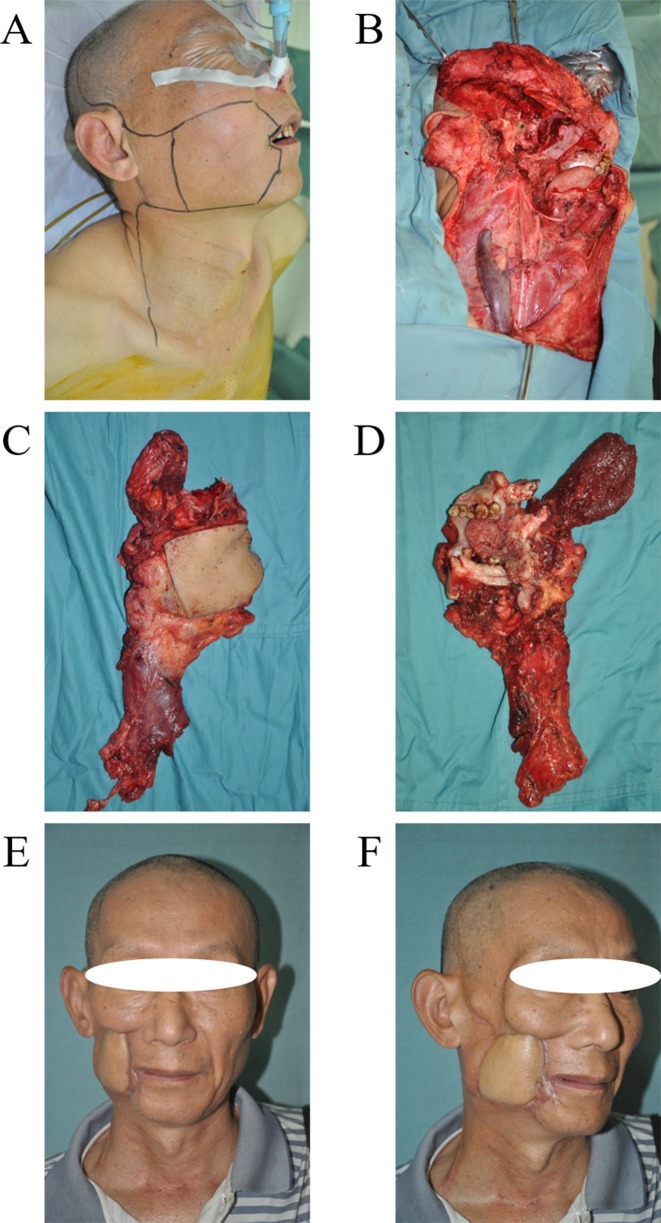
URBS of cancer located in the postgena **A**. Tumor incision design. **B**. The surgery area after tumor resection and neck dissection. **C**. The primary tumor and neck dissection tissue (from skin). **D**. The primary tumor and neck dissection tissue (from mucosa), the main body of the tumor is located in the anterior border of masseter. **E**. The photo of a patient 4 years postoperatively. **F**. The profile of patient 4 years postoperatively.

**Figure 6:**
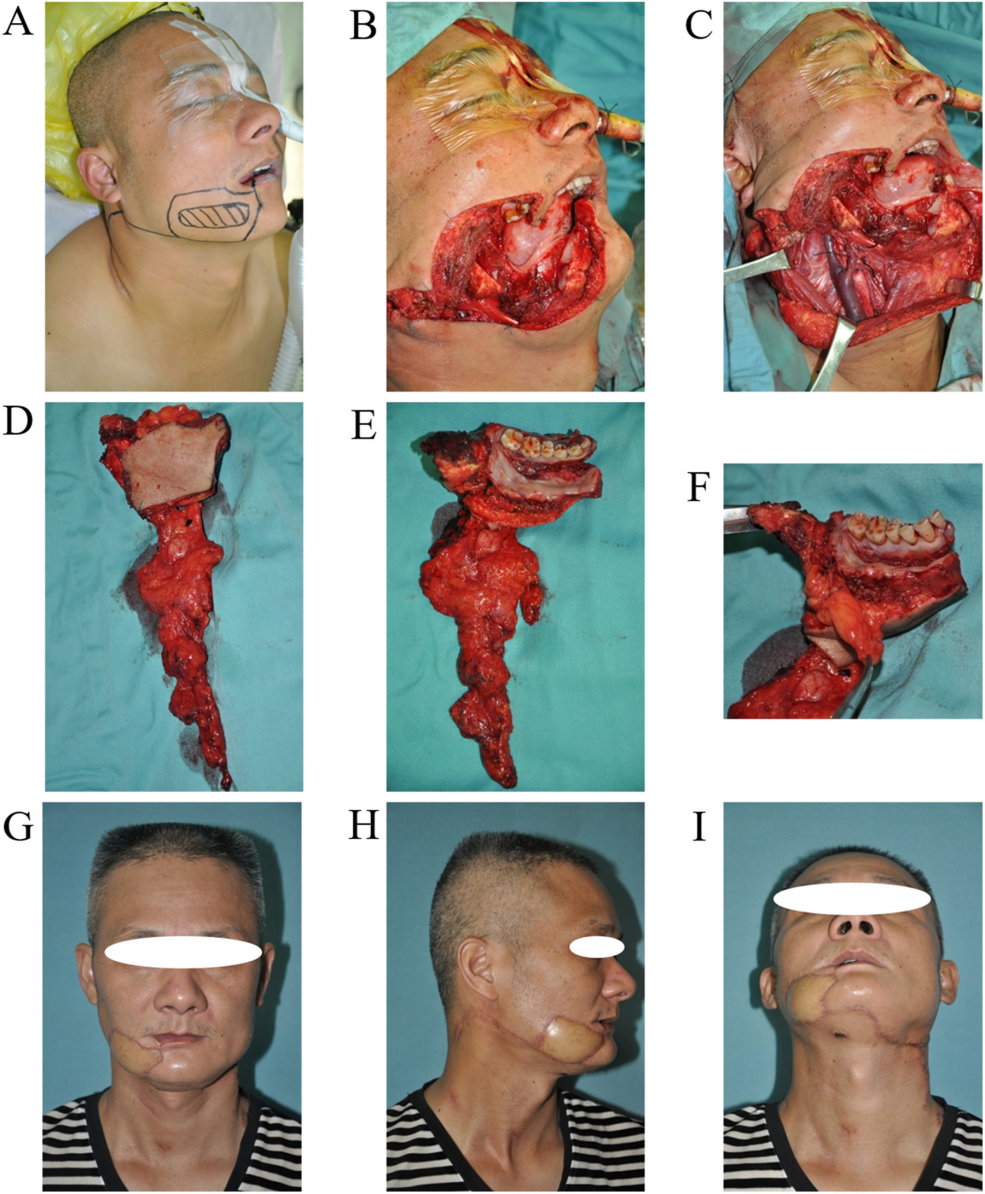
URBS of buccal cancer located below the line of occlusion **A**. Tumor incision design. **B and C**. The surgery area after tumor resection and neck dissection. **D and E**. The primary tumor and neck dissection tissue. **F**. Buccal fat pad could prevent tumor infiltrating upward. **G and I**. The photo of patient 2 years postoperatively, the incision scar was hidden.

By reviewing the preoperative imaging results and studying the postoperative tissue anatomy of patients with buccal carcinoma (Figure 3), we concluded that: the traditional surgical approach for the removal primary buccal carcinoma is not thorough enough, which is the main reason for the high rate of recurrence rate. As such, we suggest removing buccal carcinoma by cutting off the complete anatomical units. The basic concept for unit resection is the removal of the entire anatomical unit (or subunit) in which the tumor is contained rather than removing tumor with a 1-2cm histopathological margin. Starting and ending points of maxilla and mandible, temporalis muscle, pterygoid muscle, masseter, buccinator and other muscle, peripheral space of masticatory muscle and skin overlying buccal mucosa that are mentioned above are the main anatomical landmarks of URBS. URBS is performed in accordance with the following rules: 1. The primary tumor is located on the pregena: for specific anatomical features of the pregena, the cases in which the primary tumor is located in the pregena (including early invasive carcinoma), should be treated by thorough resection, that is, take extended resection together with the overlying skin. The reason is that pregena is loose and thin, which facilitates tumor invasion into the subcutaneous tissue. Resections that includes skin helps ensure adequate extension of tumor resection. This surgical method ensures enough depth while avoiding tumor disruption, and removal of complete anatomical units implements the principle of non-tumorbetter, and reduces the risk of recurrence. Tumors located on the pregena often are very close to or involve the medial mucosa of the lip, so surgical resection can not deliberately retain the upper lip, lower lip and vermilion or skin and tissue on the upper lip and lower lip for purely an aesthetic result as it is likely to result in local recurrence (Figure 4). 2. The primary tumor is located on the postgena: tissues in this area are thick. Submucosa, buccinator, buccopharyngeal fascia, buccal fat pad, medial pterygoid, mandibular ramus, masseter muscle, subcutaneous loose connective tissue and skin were included in this area. For the primary buccal carcinoma only located at the back part of the anterior border of the masseter, a non-thorough resection is usually taken to retain the skin. Tumor cells in this area readily spread along fibers of temporal muscle, medial pterygoid, masseter, and pterygomandibular space and invade the region under the zygomatic and pterygopalatine fossa. Therefore, resection of tumor in this area should attach great importance to deal with the medial pterygoid, temporalis, masseter, buccal fat pad, maxillary posterior region and mandibular ramus (inferior zygomatic, inferior temporal dissection). Therefore, extended resection of the inferior zygomatic tissue is essential for the complete removal of cancerous lesions in the postgena. When the tumor spreads across the pterygomandibular ligament to invade soft palate, all soft palate muscle of the affected side should be excised. Patients with tumor size over T3 should undertake whole parotid lobe resection as they have a higher risk of parotid lymph node metastasis. If the tumor does not invade the facial nerve, the temporofacial branch could be retained. If the tumor has spread from the postgena to the pregena, the pregena region corresponding to tumor should be removed by “through and through resection” (Figure 5). 3. The primary tumor is located below the line of occlusion: tissue in this area is less thick, and often needs through and through resection as the tumor has often invaded the mandible. Mandibular marginal resection or segmental resection should be decided according to the specific situation. For primary tumors located on the postgena below the line of occlusion, where the tumor does not make contact with the temporalis muscle and the pterygoid muscle anatomically, there is not easy to spread to the inferior zygomatic tissue, and no need for expanded resection of the inferior zygomatic region (Figure 6). 4. The primary tumor is located above the line of occlusion: resection for primary tumor above the line of occlusion or posterosuperior gingival carcinoma that invades the cheek is the same as for postgena cancer. Attention should be paid to the inferior zygomatic region and to the posterior of the maxilla. If the tumor is small and only located in the anterior of the cheek, the resection was made with reference to previous pregena cancer resection. It is worth emphasizing that resection for invasive carcinoma, even early invasive carcinoma, occurring at the pregena often must cut through the pregena skin, otherwise there is a high risk of relapse due to a lack of enough secure boundary.
